# P-1891. What’s the Reason for the Consult? Identifying Clusters of Infectious Disease Consultations in an Academic Health System

**DOI:** 10.1093/ofid/ofae631.2052

**Published:** 2025-01-29

**Authors:** Michael E Yarrington, Alison G C Smith, Arthur W Baker, Gary M Cox, Kristen Dicks, John Engemann, Patricia Kohler, Ahmad Mourad, Rasha Raslan, Wil L Santivasi, Nicholas A Turner, Rebekah Wrenn, Jason E Stout

**Affiliations:** Duke University Health System, Durham, North Carolina; Duke University, Durham, North Carolina; Duke University School of Medicine, Durham, North Carolina; Duke University Medical Center, Durham, North Carolina; Duke University Health System, Durham, North Carolina; WakeMed, Cary, North Carolina; Duke University Division of Infectious Disease, Durham, North Carolina; Duke University School of Medicine, Durham, North Carolina; Duke University Hospital, Durham, North Carolina; Duke University School of Medicine, Durham, North Carolina; Duke University Medical Center, Durham, North Carolina; Duke University, Durham, North Carolina; Duke University School of Medicine, Durham, North Carolina

## Abstract

**Background:**

In many institutions, infectious diseases (ID) consultations are requested via electronic order entry and the reason for consultation is included as free text in the order. We employed an unsupervised clustering algorithm to determine whether the consult order text can be used to ascertain clinically meaningful groups among patients for whom ID consultation is requested.

**Figure 1.**
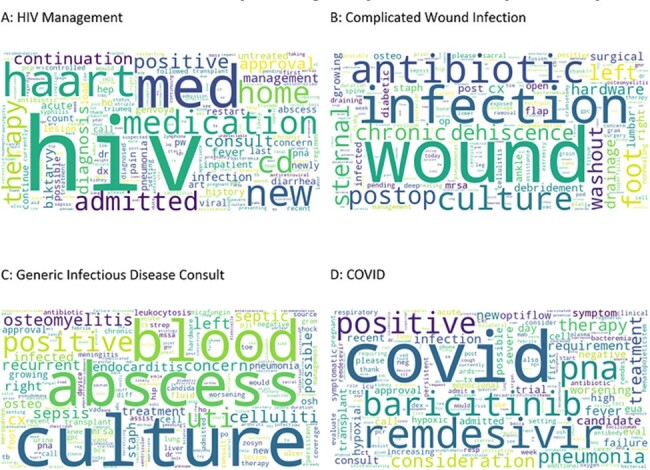
Example Word Cloud ‘Clusters’ of Free-Text Reasons for Infectious Disease Consultation and Corresponding Subject Matter Expert Interpretation

**Methods:**

We examined the initial ID consultation for all Duke University Health System inpatients receiving consultation from 1/1/2014 - 12/31/2023. The free text “Reason for Consult?” field in the consultation order was extracted, transformed into a weighted vector format using Term-Frequency Inverse-Document Frequency, and categorized using an unsupervised K-means clustering algorithm; Algorithm parameters were adjusted to optimize clinical relevance of the clusters. Within each patient cluster, we evaluated mortality and hospital length of stay, with patients censored at their time of last contact with the health system.

**Figure 2.**
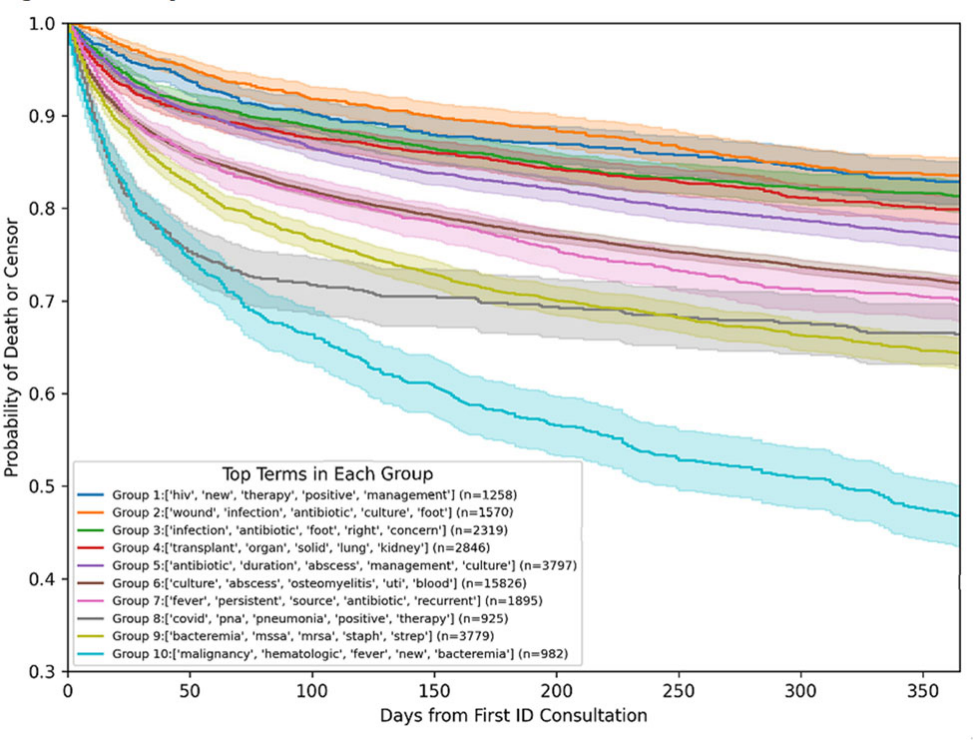
Kaplan-Meier Survival Estimates for Each Cluster

**Results:**

37,848 unique patients with an ID consultation during the study period were evaluated, and 35,197 had free-text reasons associated with the electronic consult order. A combination of the K-means Elbow Method, silhouette score, and subject matter expert evaluation identified that approximately 10 clusters were optimal to categorize the dataset. Word clouds were generated for each cluster (Figure 1) and expert assessment identified the central theme of each cluster (Table 1). We identified unique patient characteristics or syndromes in nine of the patient clusters, while the final cluster was characterized as a ‘generic’ ID consult. One-year mortality varied according to cluster (Figure 2) with the highest mortality seen in the ‘Hematologic Malignancy’ category, and lowest mortality in the ‘Complicated Wound Infection’ and ‘HIV Management’ categories.

**Conclusion:**

An unsupervised clustering algorithm allowed for the identification of clinically meaningful groups of ID consultations. If validated in other settings, this approach could present an accessible way to understand qualitative and prognostic trends in ID clinical practice.

**Table 1.**
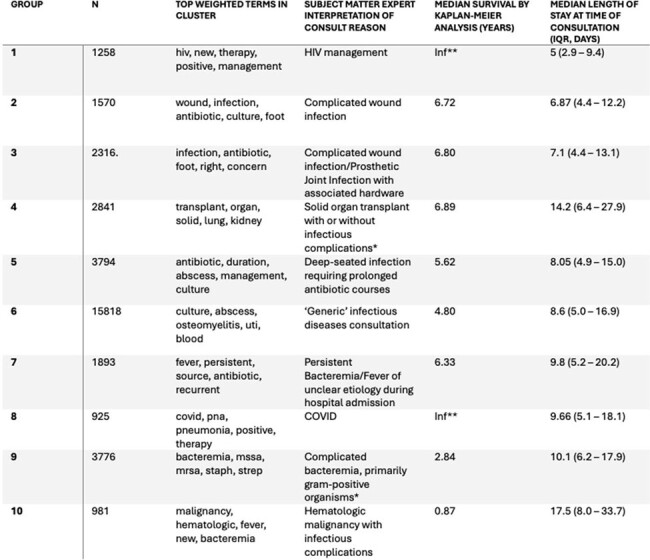
Cluster Characteristics *Institutional policy at the end of study period requires Infectious Disease consultation for patients with HIV, new lung transplant recipients, patients with candidemia or Staphylococcus aureus bacteremia, and patients for whom restricted antibiotics are requested. **Infinite median survival times indicate that the Kaplan-Meier survival curve did not cross the 50% threshold through the evaluated period.

**Disclosures:**

Kristen Dicks, MD, UpToDate: Advisor/Consultant Nicholas A. Turner, MD, MHSc, PDI: Research contract|Purio Labs: Research contract

